# A novel method to visualise and quantify circadian misalignment

**DOI:** 10.1038/srep38601

**Published:** 2016-12-08

**Authors:** Dorothee Fischer, Céline Vetter, Till Roenneberg

**Affiliations:** 1 Institute for Medical Psychology, Ludwig-Maximilian-University, Goethestr. 31, 80336 Munich, DE.

## Abstract

The circadian clock governs virtually all processes in the human body, including sleep-wake behaviour. Circadian misalignment describes the off-set between sleep-wake cycles and clock-regulated physiology. This strain is predominantly caused by external (societal) demands including shift work, early school start times and fast travels across time zones. Sleeping at the ‘wrong’ internal time can jeopardise health and safety, and we therefore need a good quantification of this phenomenon. Here, we propose a novel method to quantify the mistiming of sleep-wake rhythms and demonstrate its versatility in day workers and shift workers. Based on a single time series, our *Composite Phase Deviation* method unveils distinct, subject- and schedule-specific geometries (‘islands and pancakes’) that illustrate how modern work times interfere with sleep. With increasing levels of circadian strain, the resulting shapes change systematically from small, connected forms to large and fragmented patterns. Our method shows good congruence with published measures of circadian misalignment (i.e., Inter-daily Stability and ‘Behavioural Entrainment’), but offers added value as to its requirements, e.g., being computable for sleep logs and questionnaires. *Composite Phase Deviations* will help to understand the mechanisms that link ‘living against the clock’ with health and disease on an individual basis.

Modern lifestyle – characterised by living predominantly inside during the day and using electrical light at night – has altered our biological, internal timing by weakening the daily contrast between light and darkness. Light-dark cycles are however the most important signal (zeitgeber) used by the circadian clock for synchronization to the 24-h day. As a consequence of these weak zeitgeber conditions, the clocks of the vast majority have delayed and clash with our social, external time (*e.g*., school/work hours)^1^,^2^. This modern syndrome (referred to as circadian misalignment^3^, chronodisruption^4^, or social jetlag^5^) has various health consequences, ranging from insulin resistance and inflammation^6^, vascular events^7^, and disturbance of cell cycle checkpoints and DNA repair^8^, to metabolic diseases^9^. Circadian misalignment is especially pronounced in workers on rotational shifts and is thought to partly be responsible for their well-documented health problems (including obesity and diabetes^10^,^11^,^12^,^13^, cardiovascular diseases^14^,^15^,^16^, and different cancer types^17^,^18^). It is often defined as sleeping at the wrong circadian time, *i.e*., as desynchrony between sleep-wake behaviour and circadian physiology, such as rhythms of body temperature, melatonin and cortisol secretion. Specifically, mistimed sleep disrupts the circadian system despite intact rhythmicity of the central pacemaker in the suprachiasmatic nucleus as demonstrated by a recent study showing that 6.4% of all transcripts were expressed in a circadian manner when subjects slept in phase with melatonin secretion (showing a near 24-h rhythm) while only 1% was rhythmic when they slept out of phase^19^.

Different definitions, assessments and quantifications of circadian misalignment across studies prevent consistent and systematic investigation of its causes and consequences. To understand the link between disease and circadian misalignment, we need to reliably quantify mistimed sleep. Here, we propose a novel and simple method that can be applied beyond the sleep-wake cycle to all time series, *i.e*., any circadian output, be it physiology or behaviour. We show the potential scope and versatility of this method by using it in different populations, namely day and shift workers.

## Results and Discussion

### The Procedure

**Figure 1:**
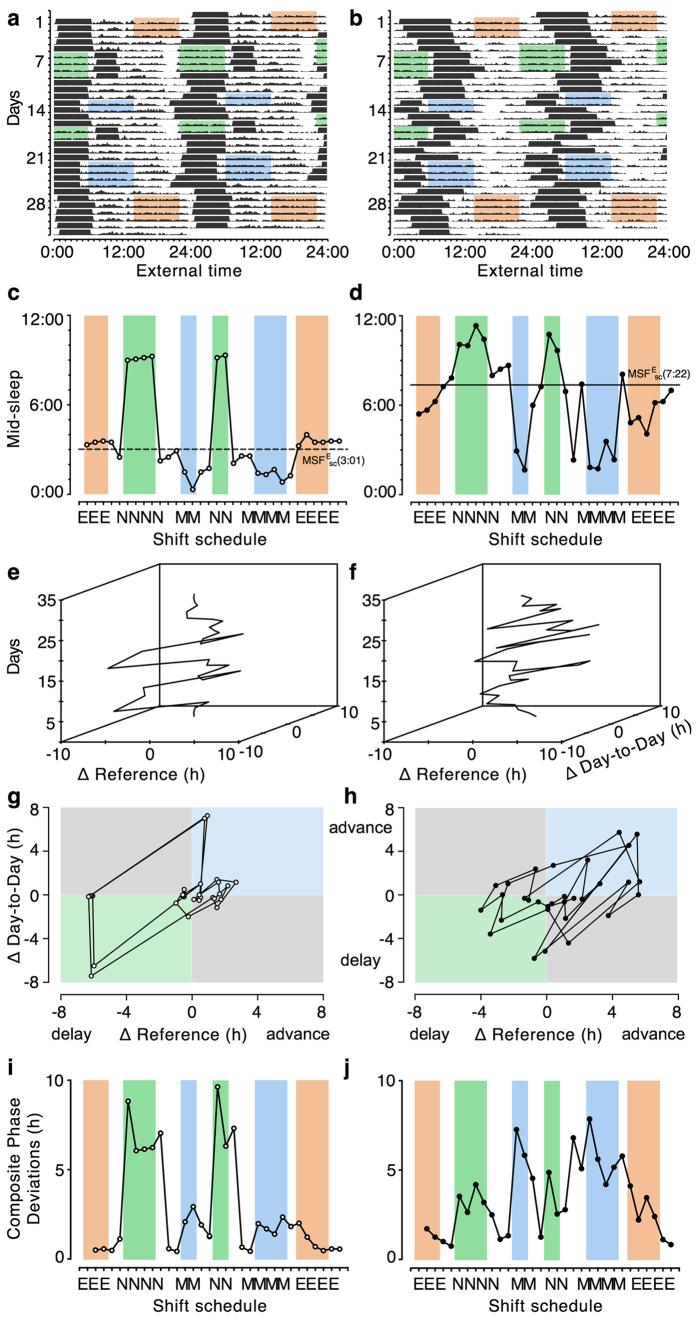
Composite Phase Deviations in shift workers. Double-plots of activity over 4 weeks of an early chronotype (MSF^E^_sc_ = 3:01; panels on the left) and a late chronotype (MSF^E^_sc_ = 7:22; panels on the right). Black horizontal bars in panels a and b represent sleep bouts. Blue shading represents morning shifts (6 a.m.–2 p.m.), orange shading evening shifts (2 p.m.–10 p.m.), and green shading night shifts (10 p.m.–6 a.m.). Profiles in panels c and d represent mid-sleep times extracted from activity recordings (horizontal lines indicate individual chronotype, MSF^E^_sc_, based on activity-derived sleep times). Panels e,f show three- and panels g,h show two-dimensional ‘Δplots’ (see text for details) illustrating advances and delays throughout the shift schedule. Note that ΔReference in these examples refers to the difference between a given mid-sleep and the individual chronotype (MSF^E^_sc_); the latter is used as the reference measure but can be replaced by other variables assumed to reflect an optimum. Panels i and j show Composite Phase Deviations (vector lengths) calculated from Δplots.

Circadian misalignment occurs between an internal, circadian time window for optimal sleep and an external time window that limits the opportunity to sleep (*e.g*., due to work or school times). The timing of the internal sleep window can be estimated by *chronotype*-measures based on an individual’s daily sleep-wake timing^20^. The Munich ChronoType Questionnaire^1^ calculates chronotype as the mid-point of sleep on work-free days and corrects it for sleep loss on workdays (MSF_sc_). Sleep mid-points have been shown to correlate well with dim light melatonin onset^21^,^22^,^23^. In shift workers, chronotype is calculated from mid-sleep on work-free days after evening shifts (MSF^E^_sc_), which interfere the least with circadian sleep times^24^. We illustrate our approach using sleep times in shift workers with the help of two very different chronotypes (MSF^E^_sc_ of 3:01 and 7:22, respectively; note that we calculated chronotype from objective activity recordings), who work identical shift schedules (Fig. 1, panels a,b).

The daily mid-sleep profiles extracted from activity recordings show in Fig. 1(panels c,d) how sleep adjusts to work times and thereby produce large day-to-day variation; they also demonstrate the strong influence of chronotype on individual sleep timing even during shift work: late chronotypes (right panels) are generally delayed in their sleep times relative to early types (left panels). If they are not submitted to drastic external changes like shift schedules, entrained rhythms normally maintain a stable phase relationship to their zeitgeber, so that sleep occurs more or less at the same time each day. However, the rotation of shift schedules chronically misplaces sleep away from the ‘window’ provided by the circadian clock. These chronic misplacements are associated with two daily discrepancies: how far away does sleep occur from its circadian window and how different are the sleep times compared to those on the previous day. Using daily mid-sleep times and chronotype (determined by mid-sleep on work-free days after evening shifts, MSF^E^_sc_), they can be quantified for any given day *i* by the following equations:


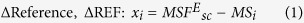



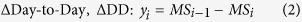


*MSF*^*E*^_*sc*_ = Chronotype (mid-sleep on free days after evening shifts, corrected for over-sleep)

*MS*_*i*_ = Mid-sleep on day *i*

*MS*_*i−1*_ = Mid-sleep on previous day

Note: As is standard in the circadian field, delays are negative and advances positive.

ΔReference in this example refers to the difference between a given mid-sleep and the individual chronotype (MSF^E^_sc_); the latter is used as the reference measure (‘circadian sleep window’) but can be replaced by other variables assumed to reflect an optimum. In Fig. 1(panels e and f), ΔReference (∆REF) and ΔDay-to-Day (∆DD) are plotted against each other with subsequent days on the third, vertical axis. Alternatively, the two ∆-variables can also be collapsed as shown in panels 1 g and h (‘∆plots’), producing a circular trajectory (mainly clockwise due to forwards shift rotation). The origin in ∆plots represents the ‘ideal state’ of the system for all chronotypes (*i.e*., sleeping within one’s internal sleep window and doing so without much variation). ∆plots reveal information about the interaction between work schedules and individual sleep times. For instance, early chronotypes show large delays at the transition to night shifts relative to both their chronotype and their previous sleep times (negative ∆REF- and ∆DD-values in the lower left quadrant). For subsequent night shifts, ∆DDs are small, while ∆REFs remain high (close to the negative arm of the x-axis). In contrast, when early types sleep on their first day off following a night shift ∆REF is small and ∆DD large (close to the positive arm of the y-axis). The Composite Phase Deviations (CPD) shown in the ∆plots can be simply quantified by their vector length (see also SI, Fig. S1):


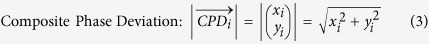


*CPD*_*i*_ = Composite Phase Deviation on day *i*

*x*_*i*_ = Distance of mid-sleep on day *i* to chronotype (MSF^E^_sc_)

*y*_*i*_ = Distance of mid-sleep on day *i* to previous day *i*−1.

We use mid-sleep times here, the method can however be applied to any variable collected as a time series (*e.g*., body temperature, reaction times or melatonin levels). Without a given individual reference (like chronotype, MSF^(E)^_sc_), data can theoretically be related to either a known optimal value or simply the (circular) average of the time series. The quantification can be calculated for single days or integrated over longer periods (sums, maxima, average of the vector lengths for, *e.g*., weeks, pre- and post comparison, etc.). Chronotype as assessed by MSF^(E)^_sc_ is a static reference, and predictions could possibly be improved when the reference is dynamically modelled, *i.e*., allowing the MSF^(E)^_sc_ to change. Such dynamics could be based on recorded light levels. However, we would not expect our results to change drastically given that all schedules in this study involved fast rotations (*i.e*., less than a week on the same type of shift) and thus, we would expect circadian phase to remain rather stable.

Using CPD vector lengths for quantification allows comparisons between and within individuals and reproduces published findings^25^: sleep timing in early chronotypes is maximally misplaced after night shifts while this occurs before morning shifts in late types (Fig. 1, panels i and j; see also SI, Fig. S2a–f, for correlation analysis). We furthermore examined the relationship between daily CPD values and self-reported sleep quality (ranging from 1 = very poor to 10 = very good), revealing CPD as a significant predictor: the higher CPD (greater misalignment) of a particular sleep bout, the worse its perceived sleep quality (b = −0.22, t = −14.88, for full model, see SI, Table S1).

### Comparison with other measures of circadian misalignment

We compared CPDs with two other measures of circadian misalignment in the shift work sample (n = 53). The Inter-daily Stability (IS^26^) calculates the stability of a rhythm based on the Sokolove and Bushell χ^2^ periodogram, a method that determines the period length of a time series^27^, while ‘Behavioural Entrainment’ (BE^28^) quantifies the overlap between light exposure and activity by computing phasor magnitudes of circular cross-correlations (cross-correlating activity and light results in a sinusoidal curve of increasing and decreasing correlation strength; the curve’s amplitude is encoded by a phasor and reflects the extent to which both time series overlap). CPDs correlated significantly with both measures (r = −0.56_IS_/−0.48_BE_, P < 0.001); higher CPD values were associated with less stable activity rhythms and less light/activity-overlap (see SI, Fig. S2a). We also analysed correlations of CPD, IS, and BE with individual chronotype revealing good agreement for CPD and IS: the later the chronotype, the greater the misalignment (r = 0.37_CPD_/−0.63_IS_, P < 0.001; see SI, Fig. S2b). No significant relationship was found for chronotype and BE (r = 0.05, P > 0.05) suggesting that opposite chronotypes can largely differ in their day-to-day sleep-wake behaviour but show similar degrees of BE as long as they keep being active while lights are on and stop being active when lights are off (*i.e*., work indoors and use light until they go to sleep; see SI, Fig. S2c,d).

IS has been successfully applied in various clinical settings with patients suffering from dementia where fragmentation of activity patterns frequently occurs^29^,^30^, and BE was shown to reflect mistimed rhythms in night shift workers^28^. Although CPD, IS, and BE produce similar results, they have different requirements. Both IS and BE need frequent, regular data sampling (hourly or even shorter intervals) for period analyses and cross-correlations, respectively, while the CPD method presented here requires only one or few value(s) per day (although we have calculated mid-sleeps from activity recordings, which also rely on frequent sampling, similar results can be achieved using sleep-logs, which need only sleep begin and end, that can be translated into mid-sleep times). In addition, the BE method relies on light levels, which are difficult to assess accurately in real-life with devices either being covered by clothes, not permitted for safety reasons, or light being recorded at the wrist-level (as in this study), which corresponds but is not identical to light exposure at the eye level. Importantly, all three measures CPD, IS and BE reflect misalignments of the sleep-wake cycle and rest-activity rhythm, respectively, and do not necessarily reflect physiological rhythms more directly regulated by the SCN.

### The shape of sleep: Islands and Pancakes

#### Shift Workers

**Figure 2:**
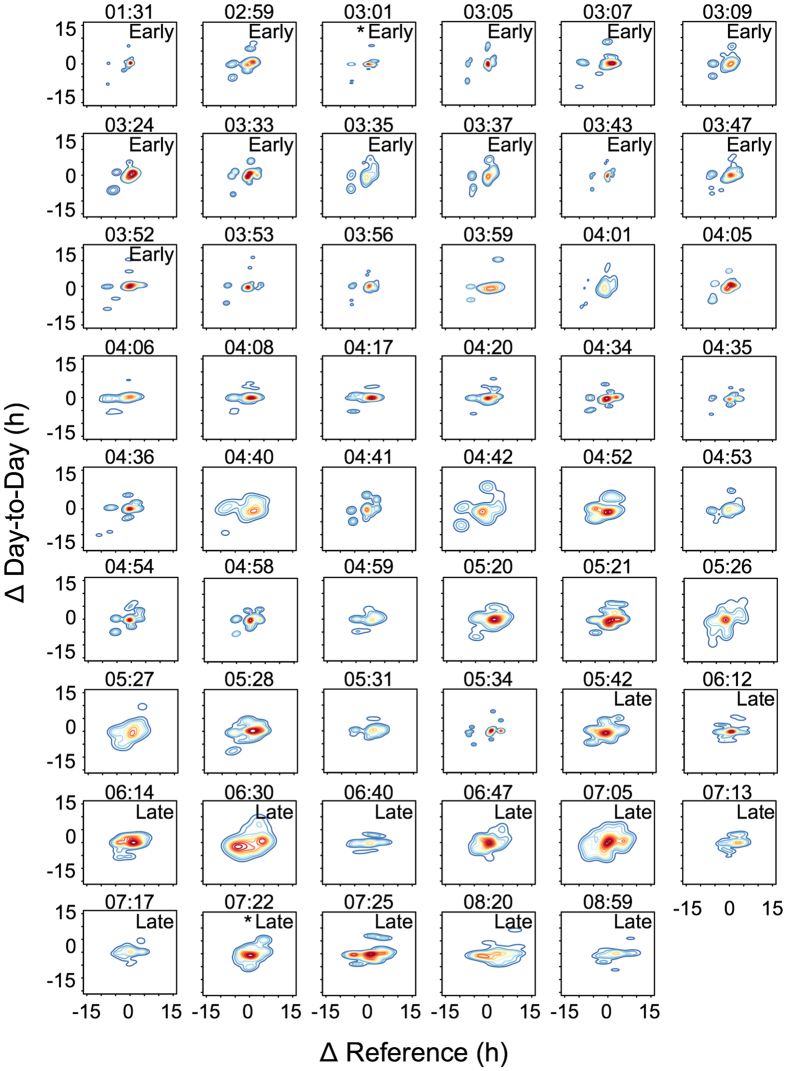
Density plots of shift workers ranked by chronotype (MSF^E^_sc_). Panels labelled “Early” represent the earliest quarter of chronotypes (n = 13, MSF^E^_sc_ < 3:53), and those labelled “Late” the latest quarter within the sample (n = 13, MSF^E^_sc_ > 5:36). Individual corrected mid-sleep times (MSF^E^_sc_) are shown above each panel as local time. Asterisks mark the two individuals shown in Fig. 1. Colour codes range from dark blue (lowest density) to dark red (highest density). Note that the last intermediate chronotype (MSF^E^_sc_ = 05:34) had a short recording period of 14 days possibly explaining the fragmented shape due to small number of data points. Mid-sleeps were extracted from activity recordings (see Methods for details). ΔReference refers to the difference between a given mid-sleep and the individual chronotype (MSF^E^_sc_); the latter is used as the reference measure but can be replaced by other variables assumed to reflect an optimum.

The ∆plots of the two shift workers (Fig. 1, panels g,h) yield differences beyond the relative timing of sleep: the early chronotype produces a relatively rigid shape with few distinct centres outside the cloud of dots near the origin. In contrast, the dots are widely scattered for the late type. To better illustrate these shape differences, we used ‘density plots’, which show contours that border areas of equal density (similar to geographic altitude maps). The area of these density plots represents the extent of misalignment and their shape the behaviour’s variability: disconnected shapes (islands) indicate less variability as most mid-sleeps either cluster near the origin or in few isolated regions, while continuous shapes (pancakes) point to more variability given that mid-sleeps scatter largely despite the regularity of the work shifts. Density plots are consistently ‘island’-shaped for early chronotype shift workers and gradually turn into ‘pancakes’ with increasing lateness (Fig. 2). ‘Islands’ can however occur in very late chronotypes when morning shifts are similarly strenuous for them as night shifts for early chronotypes (emerging in the upper right quadrant of the plot, advances).

The differences in shapes are not associated with sleep duration. Using how long and variable in duration each shift worker slept on average as predictors of CPD in a multiple regression model revealed no predictive power, and thus did not account for the shape differences (β = 0.02 and 0.16, P > 0.05, see SI for more details, Fig. S3 and Table S1). These results did not change when we replaced average sleep duration per bout with the average sleep duration per 24 h including naps (β = 0.13, P > 0.05).

It has been proposed that late chronotypes are more flexible in their sleep times than early types^31^,^32^. Chronotypes seem to differ in both, degree and regularity of light exposure, suggesting exogenous causes behind this observation^2^,^33^. Support for endogenous causes comes from a study chronotyping mice via median of activity (MOA)^34^. The authors used the Q_p_ statistic^35^, an index of oscillatory stationarity almost identical to IS calculated here, and found that mice with a later MOA had more variable activity-rest rhythms. Reduced amplitude of the circadian oscillator could account for higher variability of behavioural rhythms via greater capacity to change circadian phase, e.g., due to changing light conditions. Overall, studies modelling empirical data on human chronotypes show that reducing the circadian amplitude thereby mimicking a weaker oscillator leads to phase advances^36^,^37^. However, in none of these models did a weaker oscillator result in greater cycle-to-cycle variation of sleep-wake behaviour, leaving it still open what causes the higher variability observed in late types. We add here that it does not seem to result from differences in sleep duration, further underlining that potentially adverse effects of mistimed sleep and sleep deprivation are intertwined but not redundant.

#### Day workers

**Figure 3:**
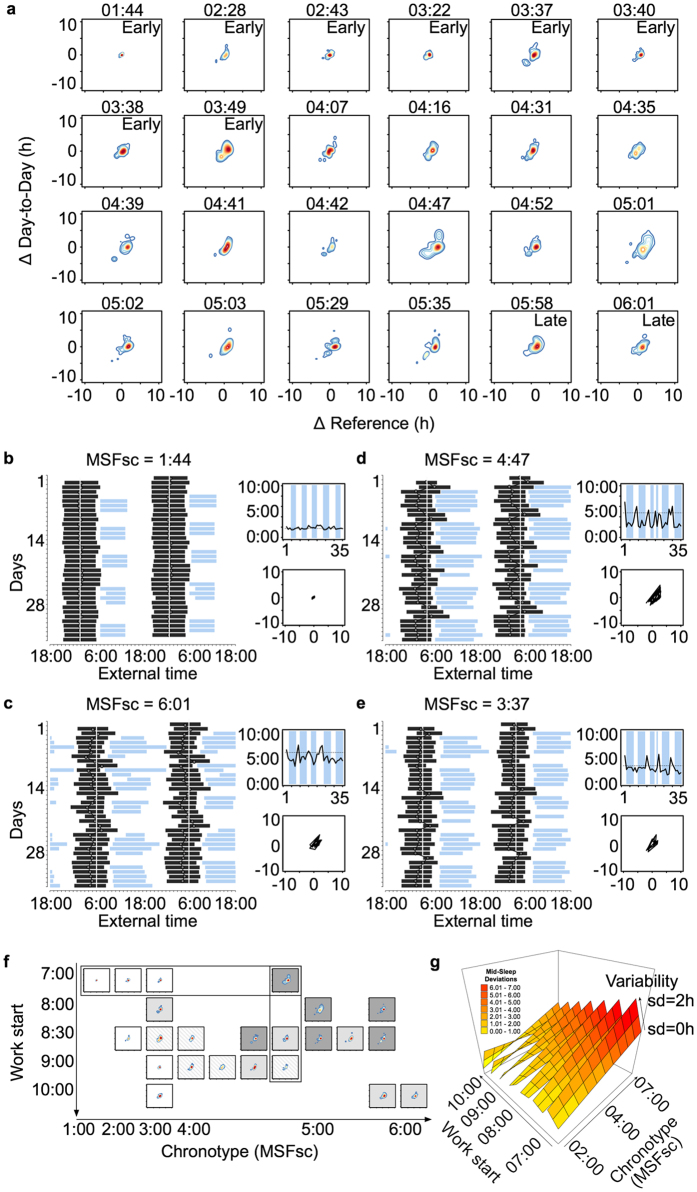
Composite Phase Deviations in day workers. Panel a shows individual density plots sorted by chronotype (see MSF_sc_ above each plot). For comparison, cut-off values for chronotype categories ‘early’ (n = 8, MSF_sc_ < 3:53) and ‘late’ (n = 2, MSF_sc_ > 5:36) are the same as for the shift workers shown in Fig. 2. Note that sleep times are based on sleep log data in the day worker sample. The sleep-wake behaviour of four individuals are displayed in more detail in panels b–e: double-plotted sleep times (black bars; white vertical line: MSF_sc_) and work times (light blue bars) over the course of 5 weeks are shown on the left of each panel, and the corresponding mid-sleep profiles (x-axis: days, y-axis: mid-sleep, dotted line: MSF_sc_) and Δplots (x-axis: ΔREF, y-axis: ΔDD) on the right. Panel f sorts the density plots of panel a according to chronotype (x-axis) and work start time (y-axis; one day worker – MSF_sc_ = 4:07 – was excluded due to missing work-time information). Grey-scale coding reflects average CPD values based on a quartile split (white: 0–1.15, striped: 1.16–1.50, light grey: 1.51–2.00, dark grey: 2.01–2.70). Results of the mixed effects regression model are graphed in panel g using a surface plot illustrating a three-way interaction effect between start and variability of work times, and chronotype. Variability is assessed as the standard deviation (sd, in h) of work start times within each individual. Colour coding reflects average CPD values in 1 h bins (yellow: 0–1, dark red: 6.01–7). Note that ΔReference in these examples refers to the difference between a given mid-sleep and the individual chronotype (MSF_sc_); the latter is used as the reference measure but can be replaced by other variables assumed to reflect an optimum.

Rotational and night-shift work are a particular challenge for sleep and the circadian system, but day work has also been shown to put strain on workers, especially on late chronotypes^5^,^9^. Using sleep log data from a sample of 24 office workers, we applied the CPD method as described above and created density plots, observing patterns that resemble those of shift workers (Fig. 3): with increasing lateness (top left to bottom right in Fig. 3a), the shapes of the density plots change from single small masses near the origin to pancakes of increasing size towards later chronotypes. Sleep bouts, work times, mid-sleep profiles and ∆plots are shown for the earliest and the latest chronotype in Fig. 3, panels b and c. While it was the early types who produced island shapes in the context of shift work (Fig. 2), they become typical for late types in the context of normal day jobs representing transitions from the workweek into the weekend and *vice versa*. Mid-sleep times can move by more than 5 hours between Sunday and Monday, and the distance between chronotype and actual mid-sleep on a given workday can exceed 4 hours (see example in panel 3d). The shape-chronotype gradient (Fig. 3a) was occasionally ‘broken’. For example, one early type (MSF_sc_ = 3:37; Fig. 3e) produced an extensive shape with a fairly large ‘island’ in the delay-quadrant (negative values for both ΔREF and ΔDD). The subject shows late and short sleep on Fridays (2.7 h later and 2.3 h shorter than the respective weekly averages) followed by recovery sleep on Saturdays (2.4 h longer than on Fridays and 4.7 h longer than the weekly average). This ‘inverse’ social jetlag in early chronotypes (*i.e*., showing circadian misalignment on weekends instead of on workdays) has been described previously^38^.

The comparison between shift and day workers (Figs 2 and 3, respectively) indicates that the shapes of the density plots depend on both chronotype and work schedule. We therefore re-arranged the density plots according to work start times (Fig. 3f), revealing two general effects: the later the chronotype (rows from left to right), the greater the Composite Phase Deviations (based here on averaged vector lengths; r = 0.75, p < 0.001), and the later work times (columns from top to bottom), the lower CPD. The latter association is however only true for start times before 9:00 a.m. (r = 0.50, p = 0.02). The relatively low CPD values in the two latest chronotypes of the sample are due to late work times. To test this statistically, we calculated a mixed effects regression model using chronotype, and work start and variability as predictors of daily CPD (Fig. 3g). The model confirmed the effects of chronotype and work start, and revealed interactions with the variability of start times (for full model, see SI, Table S2). In general, more variability leads to higher CPD levels irrespective of chronotype and start time (b = 4.19 h difference in CPD per 1 h variation in work start). However, when early chronotypes start work late, their sleep-wake behaviour seems to benefit from more variable start times (*i.e*., becomes less disrupted, 3-way interaction, see SI, Table S2). We did not see a similar effect in late chronotypes. Given the limited sample size, the findings remain to be confirmed in a larger sample, and we can only speculate about possible causes. An explanation could be that a late chronotype who usually starts work at 8:00 in the morning will sleep longer when work starts at 10:00 and will get up earlier when work starts at 6:00, and thus the variable start times create a variable sleep behaviour. In the same scenario, an extreme early type however, will get up at roughly the same time for any work start between 6:00 and 10:00, but is adversely affected by *work end times* (interfering with early sleep onsets). Hence for early chronotypes, a higher variability including earlier start and end times might be more beneficial than starting late each day.

## Conclusions

**Figure 4:**
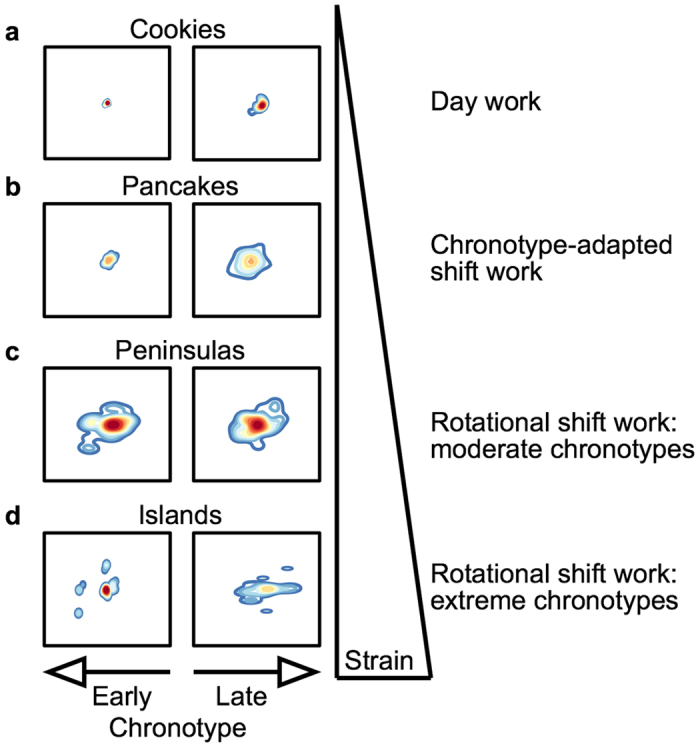
Relationship between work regime, chronotype and circadian misalignment. Depicted are density plots from individual shift workers and day workers shown before in Figs 2 and 3, respectively. Data for the chronotype-adapted shift schedule are taken from Vetter *et al*.^39^. Left panels show earlier chronotypes relative to right panels. MSF_sc_ from top to bottom and left to right: 1:44 and 5:58, 3:09 and 6:37, 5:20 and 6:47, and 3:05 and 8:59. Note that the CPD value of an island (a single dot disconnected from the rest of the shape) usually represents the maximum value within an individual, and thus the most extreme form of misalignment. However, when CPD values are averaged for an entire shape (*i.e*., across the full study period), island shapes produce generally lower overall values than pancake shapes (compare panels c and d).

Circadian misalignment occurs when circadian rhythms – including sleep – are not in synchrony with external (societal) requirements. In Fig. 4, CPD density plots illustrate the extent of this misalignment between internal time (chronotype) and external time (*e.g*., work schedules): increasing external strain (*i.e*., day work *vs*. shift work) progressively expands the CPD shape in all chronotypes until peripheral regions start to bud off the central shape (‘peninsulas’, panel c) and eventually form isolated islands under severe strain (panel d). These severe misalignments occur in extreme chronotypes in rotational shift work, where external strain increases to such an extent that sleep after the night shift (in very early types) and sleep before the morning shift (in very late types) becomes completely disconnected from their natural sleep-wake pattern (Fig. 4d). We would like to point out that, while islands usually represent the maximum CPD value of an individual (maximum misalignment), the shapes that include islands (*i.e*., panels d) generally produce lower *overall* CPD values (that is the average of the entire shape) than connected shapes of comparable size (*i.e*., panels c). CPD density plots also suggest that chronotype influences these pattern beyond the representation of internal time: late chronotypes produce generally larger shapes than early types, an indication that their sleep-wake behaviour is highly flexible allowing them to sleep at any time of day (Fig. 4, compare panels left to right). The large chronotype-differences of up to 12 h observed in humans^38^ can be used to reduce mistimed sleep in rotational shift work by aligning work time with biological time. In a recent study, we developed and implemented a shift schedule that was tailored to employees’ chronotypes^39^: while night shifts were abolished for early chronotypes, late types were no longer working morning shifts. In this chronotype-adapted shift schedule, disruption of sleep-wake behaviour was reduced close to levels of day workers (see Fig. 4a,b).

In view of an increasing number of shift workers, we need to better understand how work schedules and individual characteristics act together. Assessing exposure to shift work and describing schedules in detail is necessary but not sufficient. Although desirable, the field has so far no commonly agreed upon quantitative measure for ‘circadian misalignment’, which is more or less defined by the respective assessment method. The different existing methods will have to compete over the long run in their power to predict health effects as well as how versatile they are and how easy they are to use. Beyond the quantification via vector lengths, the Composite Phase Deviation method comes with a distinct visualisation of geometric shapes (‘islands and pancakes’). These shapes are characterised by multiple dimensions -such as variability, number of islands, expansion area- that could be used separately or combined in future studies. Future studies are encouraged to include a measure for circadian misalignment and gather more data to study the probable dose-response relationship between mistimed circadian rhythms and sleep, and health outcomes.

## Methods and Materials

### Study design

Actimetry data of shift workers were merged from three different studies conducted between 2008 and 2012 in Germany (Study 1^24^: 5/26–6/30/2008, Siemens/Cham; Study 2^24^: 9/15–10/15/2009, Siemens/Berlin; and Study 3^39^: 1/30–2/26/2012, ThyssenKrupp/Bochum). A total of 53 shift workers wore wrist-actimeters (n_1_ = 23, n_2_ = 17, n_3_ = 13) for a period of four weeks and completed sleep logs in which they reported subjective sleep quality daily on a scale from 1 = very poor to 10 = very good. Shift schedules were forwards rotating with 8-h shifts and standard transition times for morning (6 a.m.–2 p.m.), evening (2 p.m.–10 p.m.) and night shift (10 p.m.–6 a.m.) (see SI, Fig. S4 for schedule overview). Day workers (n = 24) were eligible for study participation if they worked at least three days per week, were currently not in shift work, and had no time zone travel in the previous month. They filled out sleep logs daily for five weeks between 10/19 and 11/9/2012. For a description of the shift and day worker sample, see SI, Tables S2 and S3. The Ludwig-Maximilian-University’s ethics committee and the industry works councils of Siemens AG (Berlin, Cham) and ThyssenKrupp Electrical Steel (Bochum) approved study protocols, which were conducted in accordance with ethical standards for human research. All participants gave written informed consent.

### Chronotype calculation

Chronotype was determined from actimetry or sleep log data using the mid-point of sleep on work-free days in day workers (in shift workers: work-free days after evening shifts), corrected for a potential sleep debt accumulated on workdays (MSF_sc_/MSF^E^_sc_)^1^,^24^. Cut-off values for chronotype categories were determined by the shift worker sample’s inter-quartile range (Q_25%_ = 3:53 and Q_75%_ = 5:36).

### Actimetry

Dual-axis accelerometers (Daqtix GbR, Uelzen, Germany) were used to record dynamic (motion) and static (gravity, *i.e*. change in position) acceleration from wrist-activity. An integrated sensor simultaneously measured light levels on an arbitrary scale ranging from 0–255 (no unit). Data were sampled in 1 s intervals and stored in 30 s intervals. Subsequently, data were binned into 10 min intervals for processing and analyses. If participants took off the device, they entered date and duration into a protocol, and these times were later marked as missing data.

### Sleep detection

A two-step process to detect sleep from activity data was conducted that is described in more detail elsewhere^40^. Analyses were restricted to durations between 3 h and 14 h (no naps). If a sleep bout was <3 h, it was included as main sleep if (i) it was the only one within 24 h, or (ii) it had similar sleep onsets (night shift) or offsets (morning shift) as the previous sleep bout in a block of consecutive shifts. If two sleep bouts appeared within eight hours, only the first one was included. Otherwise, sleep was imputed with average values from available data to ensure continuous series of mid-sleeps. Missing values occurred either because no sleep bout was detected following the procedures described above, or because it was marked as missing data in the participant’s protocol. In total, 37 of 1464 observations (2.5%) across 22 shift workers (41.5%) were imputed with a maximum of three missing values per person. The 24-h sleep duration considered all sleep occurrences irrespective of their length (i.e., including naps <3 h).

### Measures of circadian misalignment

The CPD method was compared with existing measures of circadian misalignment: Inter-Daily Stability (IS^26^) and ‘Behavioural Entrainment’ (BE^28^). Based on the Sokolove and Bushell *χ*^2^-periodogram^27^, IS compares each bin value and its column mean with the overall mean of the table. Their ratio becomes maximal if the time series on day *i* is exactly the same as on every following day. The ‘Behavioural Entrainment’ concept compares activity and light measures using circular cross-correlations, and phasors are calculated from the resulting functions. A phasor is a rotating vector representing the complex constant *Ae*^*iθ*^ of a sinusoidal function *y(t*) = *A sin(ωt* + *θ*) encoding amplitude and phase of an oscillation. The magnitude of a phasor is used to indicate the degree of BE reflecting how strongly light levels and activity data are related. Here, fitting the sinusoidal curve over the entire study period was not meaningful resulting in low amplitude and little between-individual variance. We therefore followed the approach by Rea and colleagues^28^ and calculated phasor magnitudes for each individual over a period of seven days including at least two night shifts. IS was computed for the complete study period in order to use all available information. Both measures were then compared with the extent of CPD for the same period.

### Data analyses and processing

Sleep detection from actimetry was performed in *ChronoSapiens* [Version 8.2]^40^,^41^. Data processing, imputation and statistical analyses were done in *R*^42^ and *STATA* (Stata/SE 12.0), and plots were drawn in Prism (*GraphPad* Software 6.0) and R using the packages *3dscatterplot*^43^ and *MASS*^44^. We conducted bivariate rank correlations and a multiple regression model to predict CPD controlling for shift schedule, age, sex, body mass index as well as number of children living in the same household. Mixed effects regression models with random effects for subjects were computed for the relationship between CPD and sleep quality in shift workers, and for the day work analysis, accounting for work start time and variability. To meet the requirements for linear regression, Breusch-Pagan tests for homoscedasticity, Shapiro-Wilk tests for normal distribution of residuals, and calculated variance inflation factors (VIF) for multi-collinearity were performed. Level of significance was set to α = 0.05. To generate density plots, we first created scatter plots showing ΔREF on the x-axis and ΔDD on the y-axis. We then used the *R* command *kde2d* from the package *MASS* for a two-dimensional kernel density estimation of the data distribution. Last, we added contour lines to the plots using the *R* command ‘contour” with default bandwidths determined via normal reference distribution.

## Additional Information

**How to cite this article**: Fischer, D. *et al*. A novel method to visualise and quantify circadian misalignment. *Sci. Rep.* **6**, 38601; doi: 10.1038/srep38601 (2016).

**Publisher’s note:** Springer Nature remains neutral with regard to jurisdictional claims in published maps and institutional affiliations.

## Supplementary Material

Supplementary Information
