# Supplementary material for: A novel method to visualise and quantify circadian misalignment
Source: Sci Rep. 2016 Dec 8;6:38601. doi: 10.1038/srep38601 (PMC5144069; doi:10.1038/srep38601)
Supplement: Supplementary Information [file srep38601-s1.pdf]

A novel method to visualise and quantify circadian misalignment

Supplemental Information

Dorothee Fischer[1,†], Céline Vetter[1,‡] & Till Roenneberg[1,*]

[1] Institute for Medical Psychology, Ludwig-Maximilian-University, Goethestr. 31, 80336 Munich, DE

[†] Present address: Harvard T.H. Chan School of Public Health, Department of Environmental Health, Boston, MA, USA

[‡] Present address: Channing Division of Network Medicine, Brigham and Women's Hospital and Harvard Medical School, Boston, MA, USA

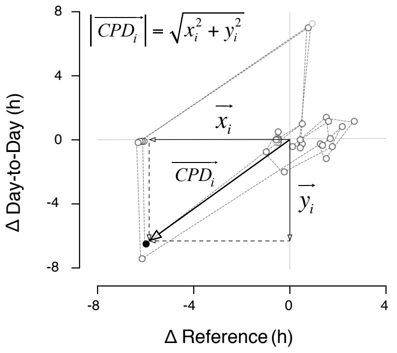

Figure S1 | Quantification of Composite Phase Deviations. Composite Phase Deviations (CPD) are quantified by the length of a two-dimensional vector using Pythagoras' theorem and represent the distance of any data point to the origin. ΔReference in this example refers to the difference between a given mid-sleep and the individual chronotype (MSF^{E}_{sc}); the latter is used as the reference measure but can be replaced by other variables assumed to reflect an optimum.

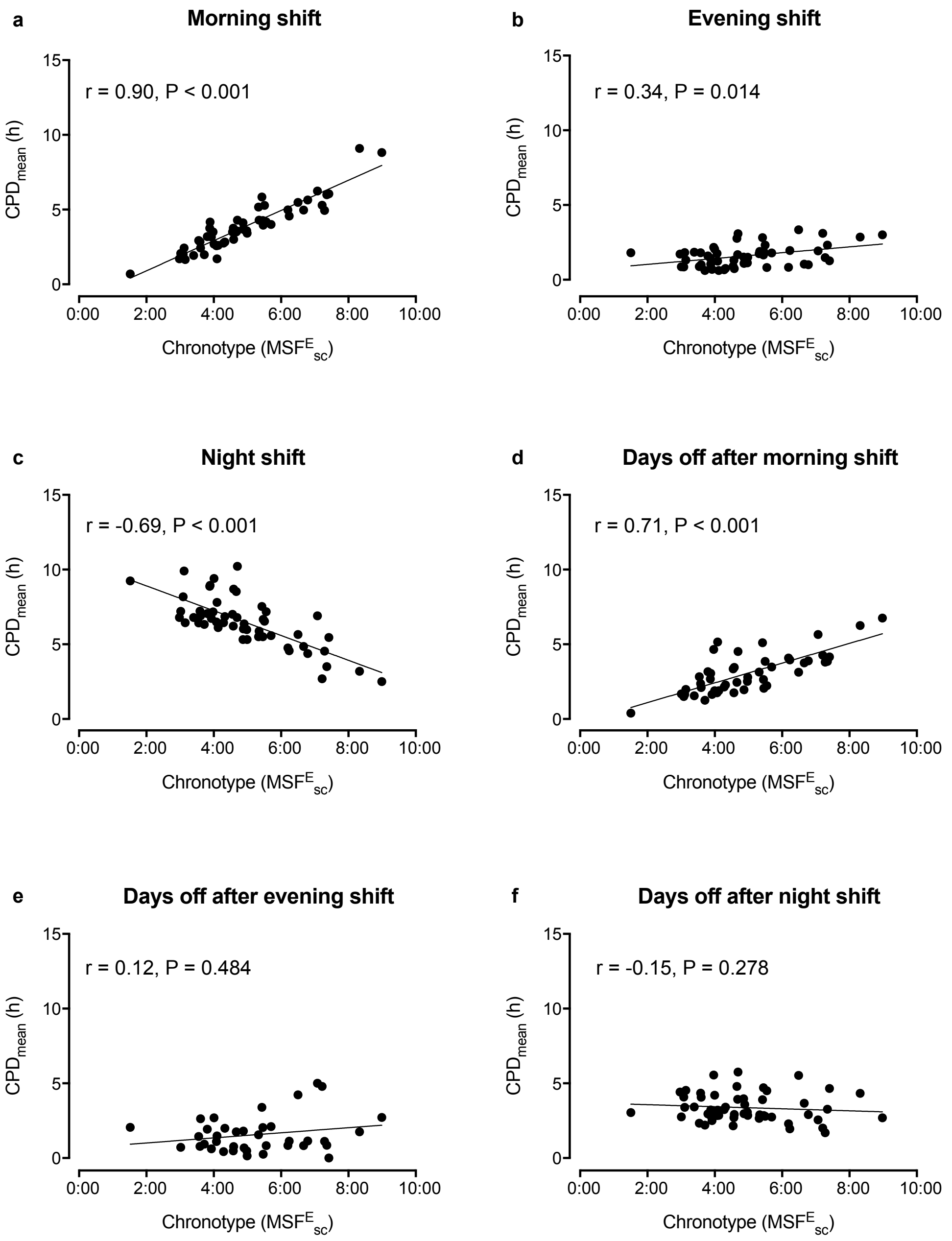

Figure S2 | Relationship between individual chronotype (MSF$^{E}_{sc}$) and Composite Phase Deviation (CPD). CPD values were averaged within each individual across all morning (a), evening (b), and night shifts (c), as well as respective the work-free days (d-f). Rank correlation analysis (Spearman rho) revealed that the later the chronotype, the higher CPD for morning shifts, evening shifts and work-free days after morning shifts, and the lower CPD for sleep after night shifts. No significant correlations were observed for work-free days after evening and night shifts.

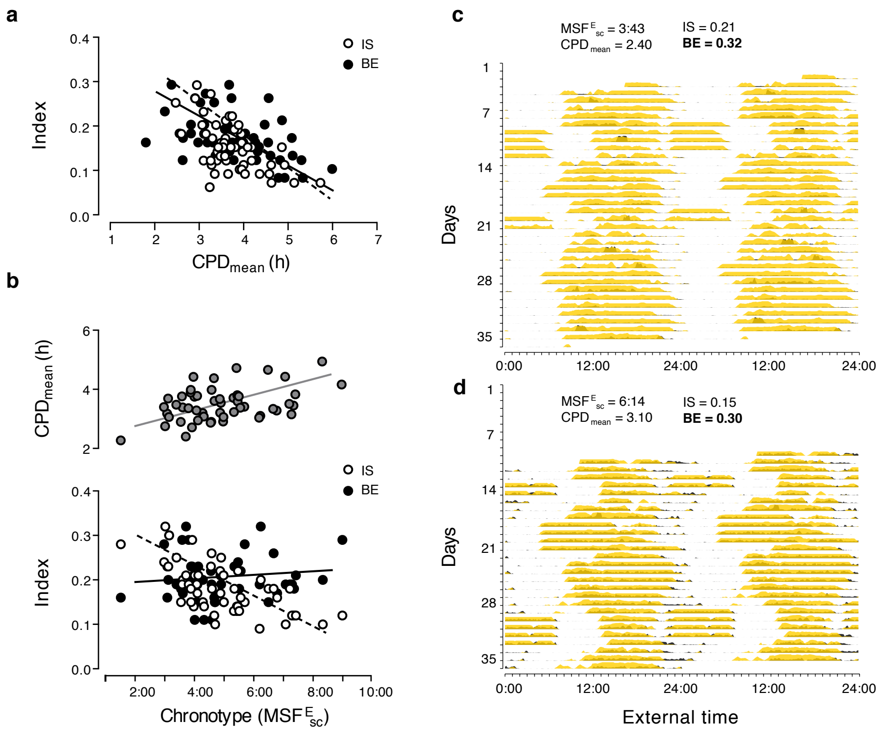

Figure S3 | Comparison of Composite Phase Deviations (CPD) with Inter-Daily Stability (IS) and 'Behavioural Entrainment' (BE) in the shift work sample (n = 53). (a) The negative relationships indicate good congruence between averaged vector lengths (CPD_{mean}) and other measures of circadian disruption as higher indices indicate lower circadian disruption in IS and BE ($r = -0.56_{IS}/ -0.48_{BE}$, $P < 0.001$). (b) Association between measures of circadian disruption and chronotype (MSF^{E}_{sc}). CPD and IS showed good correlations ($r = 0.37_{CPD}/ -0.63_{IS}$, $P < 0.001$), whereas BE did not significantly relate to chronotype ($r = 0.05$, $P > 0.05$). (c, d) Double plots for light and activity data of an early (upper panel) and a late chronotype (lower panel) working rotational shifts. BE is comparable between both types (0.32 vs. 0.30); yet, CPD and IS differ noticeably (2.40 and 0.21 vs. 3.10 and 0.15).

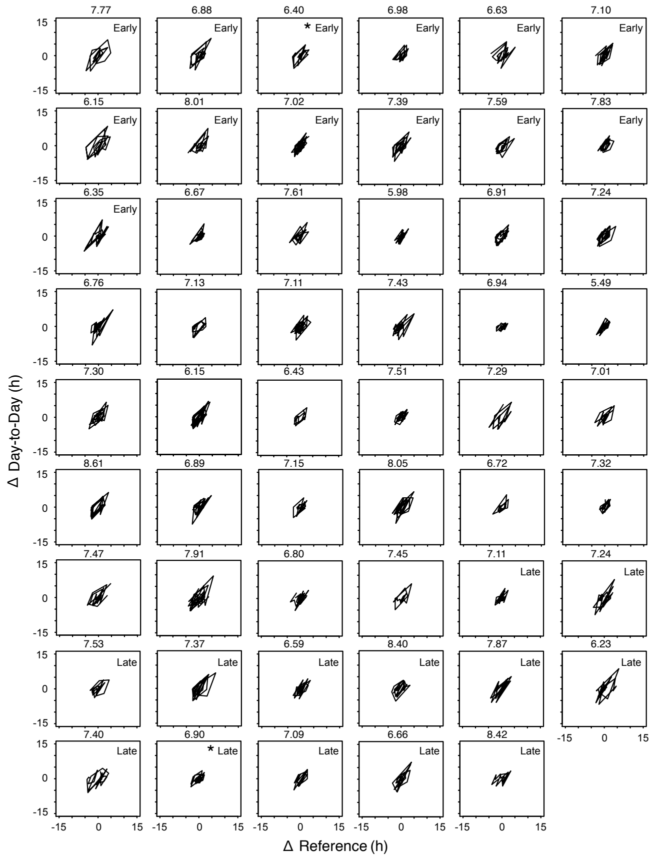

Figure S4 | Δplots for sleep duration according to chronotype in the shift worker sample. No pattern was observed that explained chronotype-shapes of mid-sleep Δplots, *i.e.,* more or less variable sleep durations were similarly distributed within and between chronotype categories. The individual sleep duration (weighted average across the study period) is shown above each panel in hours. Panels labelled "Early" represent the earliest quarter of chronotypes (n = 13, MSF^{E}_{sc} < 3:53), and those labelled "Late" the latest quarter within the sample (n = 13, MSF^{E}_{sc} > 5:36). Asterisks mark the two individuals used as examples for the approach (see main text). ΔReference in this example refers to the difference between a given mid-sleep and the individual chronotype (MSF^{E}_{sc}); the latter is used as the reference measure but can be replaced by other variables assumed to reflect an optimum.

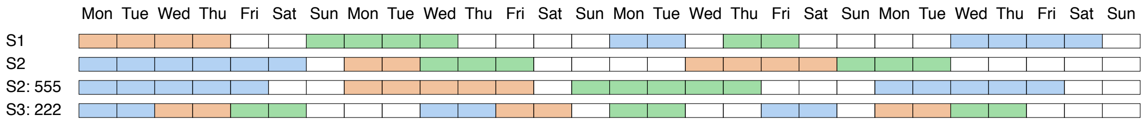

Figure S5 | Shift schedules at the different study sites in Germany. Schedule S1 refers to study site 1 in Cham, schedule S2 refers to study site 2 in Berlin (with two different schedules worked by two independent subsamples), and schedule S3 refers to study site 3 in Bochum (all study sites were in Germany). Each rotation is completed after four weeks, except for schedule S2: 555, which is completed after three weeks. Blue box = morning shift (6 a.m. – 2 p.m.), orange box = evening/afternoon shift (2 p.m. – 10 p.m.), green box = night shift (10 p.m. – 6 a.m.), white box = work-free day.

Table S1 | Mixed effects regression model predicting subjective sleep quality in shift workers

Random effects		Variance	sd	n
Subject	Intercept	1.46	1.27	53 (1,463 observations)
Residual		2.21	1.49	

Variable	Estimate	se	t
Intercept	5.04	2.75	1.83
Chronotype (MSF^{E}_{sc})	0.07	0.26	0.28
CPD_{daily} (h)	-0.14	0.02	-9.35
SD_{daily} (h)	-0.28	0.02	-14.34
Age (yrs)	-0.05	0.02	-2.02
Sex (ref: females)	-2.71	1.92	-1.41
BMI (kg/m^2)	0.07	0.07	0.98
Schedule (ref: S3: 222)			
S1	-0.17	0.20	-0.85
S2	-0.28	0.18	-2.14
S2: 555	-0.16	0.12	-1.36

Mixed model regression including daily Composite Phase Deviations (CPD_{daily}) as predictor of sleep quality. Participants reported daily sleep quality in sleep logs on a scale from 1 = very poor to 10 = very good. sd = standard deviation. n = sample size. se = standard error. Estimate = unstandardised coefficient. MSF^{E}_{sc} = mid-sleep on work-free days after evening shifts, corrected for over-sleep.

Table S2 | Regression model predicting average Composite Phase Deviations (CPD_{mean}) in shift workers

F (df)	P	R^2	Adjusted R^2	n
4.04 (10,40)	< 0.007	0.50	0.38	51

Variable	b	se	t	P > | t |	β
Chronotype (MSF^E_{sc})	0.16	0.05	3.42	0.001	0.45
SDD_{mean} (h)	0.14	0.11	1.25	0.219	0.16
SD_{av} (h)	0.02	0.12	0.13	0.900	0.02
Age (yrs)	0.01	0.008	1.56	0.126	0.24
Sex (ref: females)	0.33	0.18	1.80	0.079	0.31
BMI (kg/m^2)	0.02	0.01	1.12	0.268	0.15
Children	-0.22	0.16	-1.34	0.188	-0.20
Schedule (ref: S3: 222)					
S1	-0.32	0.20	-1.60	0.118	-0.31
S2	0.38	0.28	1.36	0.180	0.28
S2: 555	0.03	0.26	0.13	0.900	0.02
Constant	1.34	1.05	1.28	0.209	

Multiple regression model including averaged vector lengths of sleep duration (SDD_{mean}) and overall sleep duration (SD_{av}) as predictors of average Composite Phase Deviations (CPD_{mean}). We calculated the same approach using daily sleep durations instead of mid-sleeps to test whether differences in sleep duration would explain the differences in mid-sleep. Neither SDD_{mean} nor SD_{av} had predictive power ($p > 0.05$), thus not accounting for the observed pancake-shapes in later chronotypes.

Note that two participants (MSF^E_{sc} = 4:42 / 8:20) displayed outlier values (more than three inter-quartile ranges away from the sample mean) and were therefore excluded from regression analyses (n = 51). Adjusted/R^2 = effect size of overall regression model. n = sample size. b = unstandardised coefficient. β = standardised coefficient. MSF^E_{sc} = mid-sleep on work-free days after evening shift, corrected for over-sleep. SDD_{mean} = overall sleep duration deviation. SD_{av} = averaged weighted sleep duration. BMI = body mass index. se = standard error.

Table S3 | Mixed effects regression model predicting Composite Phase Deviations in day workers (CPD)

Random effects		Variance	sd	n
Subject	Intercept	0.07	0.27	23 (772 observations)
Residual		0.64	0.80	

Variable	Estimate	se	t
Intercept	-9.01	5.52	-1.63
Chronotype (MSF_{sc})	1.09	0.70	1.57
Work start time (WST, local time)	4.19	7.22	0.58
Work start variability (WSV, h)	2.97	1.32	2.26
WST*WSV	-0.62	0.76	-0.81
WST*MSF_{sc}	-0.32	0.16	-1.94
WSV*MSF_{sc}	-0.20	1.58	-0.13
WST*WSV*MSF_{sc}	0.07	0.16	0.42

Mixed model regression including work start times (WST) and variability (WSV) as predictors of Composite Phase Deviations (CPD). Participants reported daily work start times in sleep logs, and the variability of these start times was determined by the standard deviation within each individual across the study period.

Note that one participant (MSF^{E}_{sc} = 4:07) did not report work times and was therefore excluded from regression analyses (n = 23). Sd = standard deviation. sd = standard deviation. n = sample size. se = standard error. Estimate = unstandardised coefficient. MSF_{sc} = mid-sleep on work-free days, corrected for over-sleep.

Table S4 | Descriptive information of total shift worker sample and according to chronotype categories

	Total sample (n = 53)	Early (n = 13)	Intermediate (n = 27)	Late (n = 13)
Chronotype ($MSF^{E}_{sc,}$ local time, mean ± sd, range)	4:56 ± 91min	3:16 ± 37min	4:43 ± 32min	7:04 ± 0.89h
	(1:31 – 8:59)	(1:31 – 3:53)	(3:54 – 5:34)	(5:42 – 8:59)
Age (years, mean ± sd, range)	35 ± 9.61	35.31 ± 8.71	35.81 ± 10.35	33 ± 9.31
	(19 – 55)	(21 – 47)	(19 – 55)	(19 – 48)
Sex (% female)	54.72	53.85	62.96	38.46
Years in shift work	19.35 ± 8.17	19.60 ± 7.09	19.23 ± 9.69	19.40 ± 5.94
	(2 – 30)	(9 – 26)	(2 – 30)	(12 – 27)
BMI (kg/m^2, mean ± sd, range)	26.19 ± 4.90	28.79 ± 7.13	25.02 ± 3.60	26.03 ± 3.85h
	(19 – 47)	(21 – 47)	(19 – 35)	(20 – 34)
Children (% without)	60.38	46.15	74.07	58.35
Total sleep duration (hours, mean ± sd, range)	7.14 ± 0.63	6.94 ± 0.73	7.09 ± 0.64	7.29 ± 0.66
	(5.49 – 8.61)	(5.40 – 8.13)	(5.49 – 8.61)	(6.23 – 8.42)

Chronotype categories were chosen according to inter-quartile range of the sample. MSF^{E}_{sc} = mid-sleep on free days after evening shift, corrected for over-sleep. sd = standard deviation. BMI = body mass index. No significant differences are observed between early, intermediate, and late types regarding age, body mass index, and sleep duration (one-way anovas, $P > 0.05$). Groups differed with respect to chronotype ($F = 111.02$, $P < 0.001$), sex and number of participants not having children (χ^2, $P < 0.05$).

Table S5 | Descriptive information of the day worker sample

	Total sample (n = 24)
Chronotype ($MSF_{sc,}$ local time, mean ± sd, range)	4:36 ± 1.08h (1:56 – 6:10)
Age (years, mean ± sd, range)	29.42 ± 3.91 (21 – 38)
Sex (% female)	58.33 (n = 14)
BMI (kg/m^2, mean ± sd, range)	23.41 ± 5.00 (18 – 40)
Work start time (local time, mean ± sd, range)	8:40 ± 0.85h (7:00 – 10:30)
Work start variability (hours, mean ± sd, range)	0.77 ± 0.52 (0.00 – 2.10)

MSF_{sc} = mid-sleep on work-free days, corrected for sleep debt. BMI = body mass index. Work start times are based on sleep-log reports. Work start variability is assessed as the standard deviation of reported start times across the study period.
